# Supplementary material for: March Mammal Madness and the power of narrative in science outreach
Source: eLife. 2021 Feb 22;10:e65066. doi: 10.7554/eLife.65066 (PMC7899649; doi:10.7554/eLife.65066)
Supplement: Supplementary file 1. [file elife-65066-supp1.pdf]

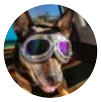

Katie Hinde @Mammals_Suck
26 Mar 19 • 27 tweets • Mammals_Suck/status/1110366004981329920

Tr

UP NEXT: 7th-seeded TIGER QUOLL versus 2nd-seed NIMRAVID #2019MMM (a collaborative narration w/ @PKurnath)

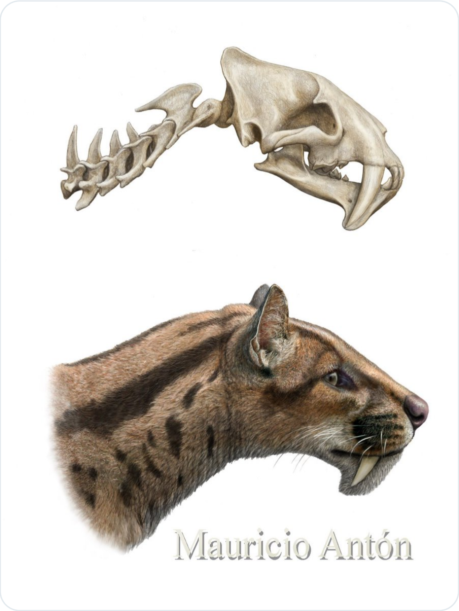

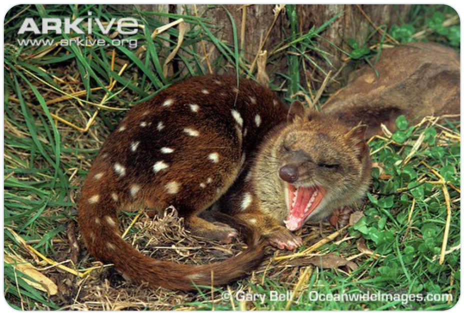

The largest carnivore on Australia's mainland today (15lbs/7kg/32 stoats), the Tiger quoll hunts like cats, only moving when the prey's head is down (Jones et al., 2001) #StoatsAsMeasurement #SneakyNotACat #2019MMM

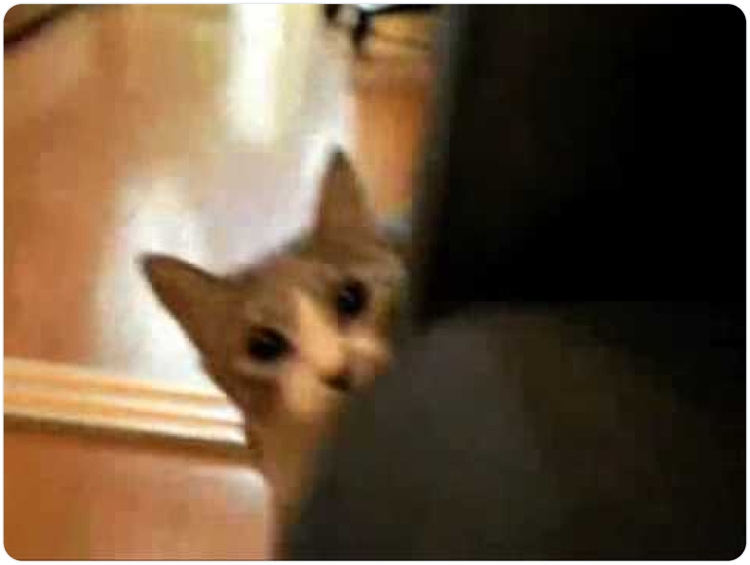

https://www.youtube.com/embed/FG6iF7fPzkU

Which is how the Tiger quoll got through round 1, by making a meal out of the Cat-

bird #YumYum #2019MMM

Our second competitor is the Nimravid, Hoplophoneus mentalis, an ancient carnivore that was more like a saber-toothed badger & likely weighed 165lbs/75kg/341 stoats #StoatsAsMeasurement #OnceThoughtACatButNotACat #2019MMM

This Nimravid is coming off a win over the DandeLION, sneezing & crushing the plant before an ancient grouse finished the plant off #2019MMM

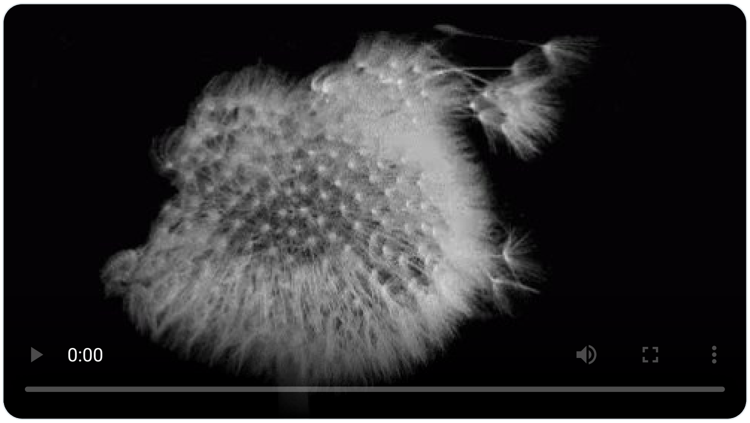

Tonight, we find both #AltFelids back 35 million years ago among the shrubs & woodlands in what today is South Dakota #BadLands #2019MMM

Tiger quoll would prefer a denser forest but its compact stature allows it move with ease through the vegetation as it looks for medium-sized mammals to munch (11lbs/5kg; Glen & Dickman, 2006; Belcher et al., 2007) #MesoPredator #2019MMM

Nimravid is also hunting, but slightly less gracefully: "Stupid, slow, & awkward though it may have been in comparison with modern cats, it was nevertheless the most active flesh-eater of the time." (Hough, 1949) #BrutallyHonest #2019MMM https://pubs.usgs.gov/pp/0221h/report.pdf

Nimravid is ragged. His left eye is puffy & swollen, his vision is obscured. As he breathes, a wheeze reveals an obstructed nasal passage. Scents rafting on the wind are now harder to detect & his wheezing betrays his ambush position... #2019MMM

...a single dandelion seed & it's whispy float, inhaled during the round one battle, have become embedded in nimravid's nasal cavity!! #TheInnocentAssasins #2019MMM https://onlinelibrary.wiley.com/doi/pdf/10.1111/j.1939-1676.2010.0580.x

Close by, a struggle then a silence... and faintest scent of death... wheezing Nimravid goes to investigate #2019MMM

Tiger quoll has just taken down an ancient hare (Palaeolagus, Wood, 1957)! Ripping strips of fur & flesh, Tiger quoll spots the creeping Nimravid #2019MMM https://www.jstor.org/stable/pdf/2406062.pdf

While wheezing, Nimravid considers the gains of stealing a meal from Tiger quoll... #Kleptoparasitism #2019MMM

THWONCK!!! #2019MMM

Suddenly, another nimravid ATTACKS our Nimravid!! During the Oligocene, the main competitor that nimravids faced were OTHER nimravids (Hough 1949) as they fought & killed each other (Boyd et al., 2013) #2019MMM

This attacking OTHER nimravid has #2019MMM Nimravid by the head!! #2019MMM

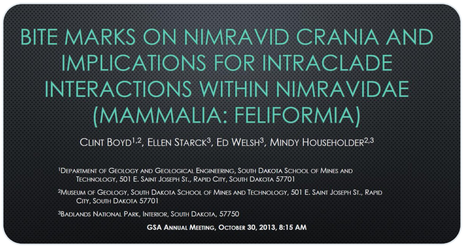

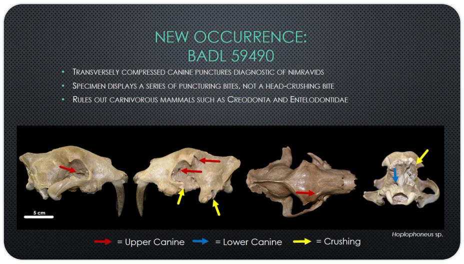

Saber-teeth puncture our #2019MMM Nimravid's skull by the right eye!!! #CARNAGE (photo by Mindy Householde)

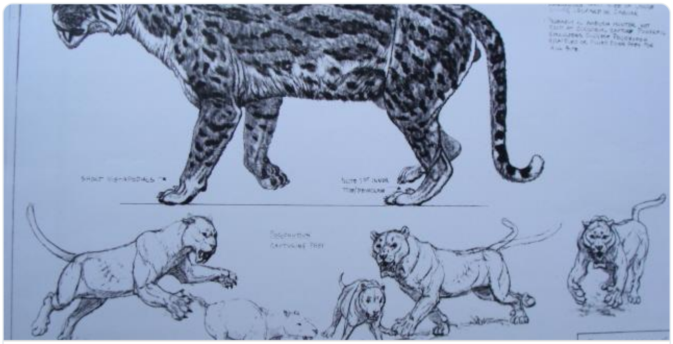

Saber-tooth-like cats ambushed and killed their own kind

Looking close at suspicious marks and cuts present in the skulls of saber-tooth like cats which roamed North America millions of years ago, paleontologist

https://www.zmescience.com/research/discoveries/saber-tooth-like-cats-ambushed-an…

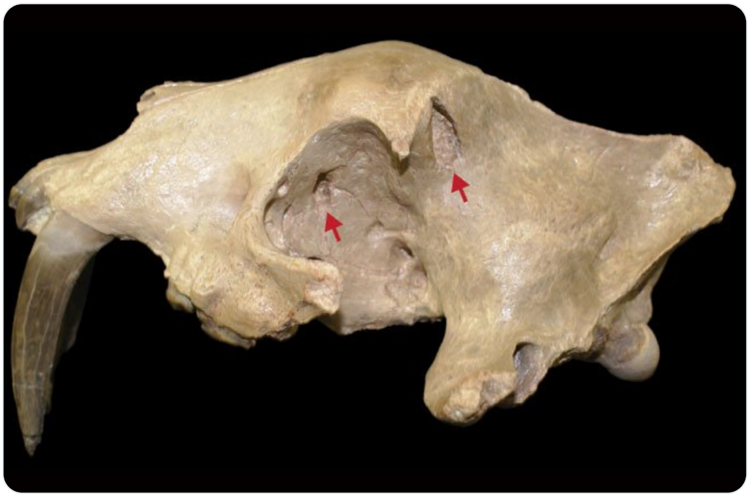

Twisting like a stout badger, #2019MMM Nimravid breaks free from the OTHER Nimravid, but with significant damage to what had been its ONE GOOD EYE!!!

#2019MMM Nimravid leaps blindly, passing the OTHER nimravid!

OTHER nimravid jump attacks #2019MMM Nimravid, sinking his saber-teeth into our #2019MMM Nimravid's back, piercing the scapula!!!

THE TWO NIMRAVIDS ARE STUCK TOGETHER!!!!! #FossilEvidence from the @AMNH's archives of the AMNH bulletin http://digitallibrary.amnh.org/handle/2246/1972 #TwistTheKnife #2019MMM

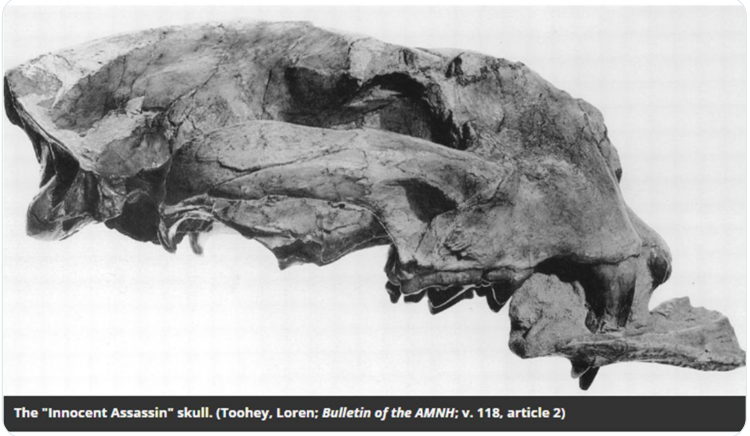
The "Innocent Assassin" skull. (Toohey, Loren; *Bulletin of the AMNH*; v. 118, article 2)

Thrashing in the mud, #2019MMM Nimravid can not twist to counter-attack; OTHER Nimravid can't extract his sabertooth where it is embedded in bone #2019MMM

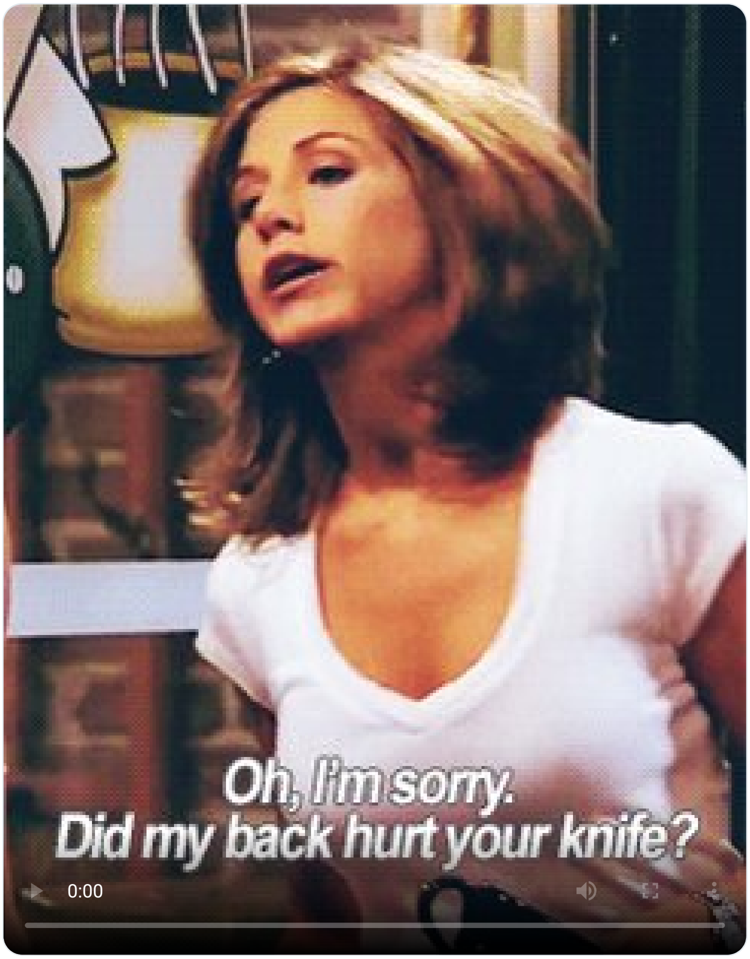

Bound in mutually assured destruction, the two nimravids slowly perish, locked

together for eternity. #Infection #Starvation #Dehydration #Predation #2019MMM

Only Loren Eiseley's poem, INSPIRED BY THE FOSSIL FIND, can do justice to a true battle so epic... excerpted below... THE INNOCENT ASSASSINS #2019MMM http://www.eiseley.org/Resources/reader/teachers/pdf/innocent-assassins-learning-supplement-lesson-05.pdf

"This was no ordinary death, though forty million years
lay between us and that most gaping snarl.
Deep-driven to the root a fractured scapula9
hung on the mighty saber undetached; two beasts
had died in mortal combat, for the bone
had never been released" -Eiseley #2019MMM

Near the doomed Nimravids, Tiger Quoll finishes his hare #2019MMM

TIGER QUOLL DEFEATS NIMRAVID!!! #2019MMM

...
