# Supplementary material for: March Mammal Madness and the power of narrative in science outreach
Source: eLife. 2021 Feb 22;10:e65066. doi: 10.7554/eLife.65066 (PMC7899649; doi:10.7554/eLife.65066)
Supplement: Supplementary file 2. [file elife-65066-supp2.pdf]

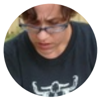

Dr. Jessica Light @je_light

14 Mar 19 • 20 tweets • je_light/status/1105990824414842880

FIRST UP: #8 seed Springhare (Rodentia: Pedetes capanesis) vs #9 seed Jackrabbit (Lagomorpha: Lepus californicus) #2019MMM

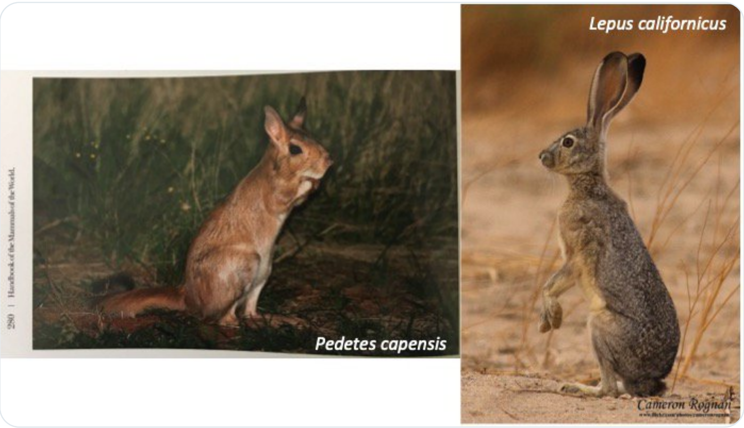

Why are these 2 species in the Jump Jump division? They're highly adapted for saltatorial, or jumping, locomotion. In fact, Springhares belong to the rodent family Pedetidae, which is Greek for "leaper or dancer" #2019MMM

Although rodents & lagomorphs are closely related (last shared a common ancestor ~80 million years ago; timetree.org), the adaptations that Springhares & Jackrabbits have for jumping evolved independently #2019MMM

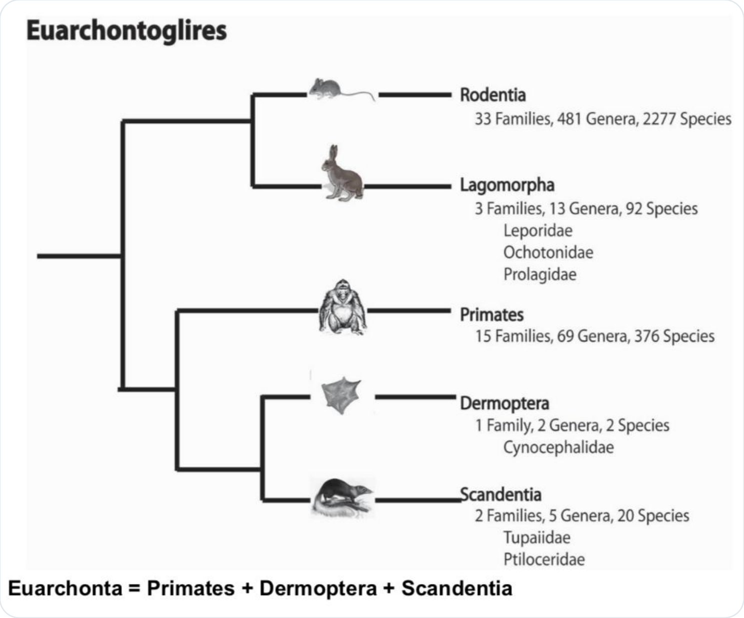

Saltatorial locomotion has evolved independently multiple times across vertebrates &

invertebrates alike (convergent #evolution), & more times in mammals than you would think. In rodents alone, this jumping locomotion is seen across several, distantly related families #2019MMM

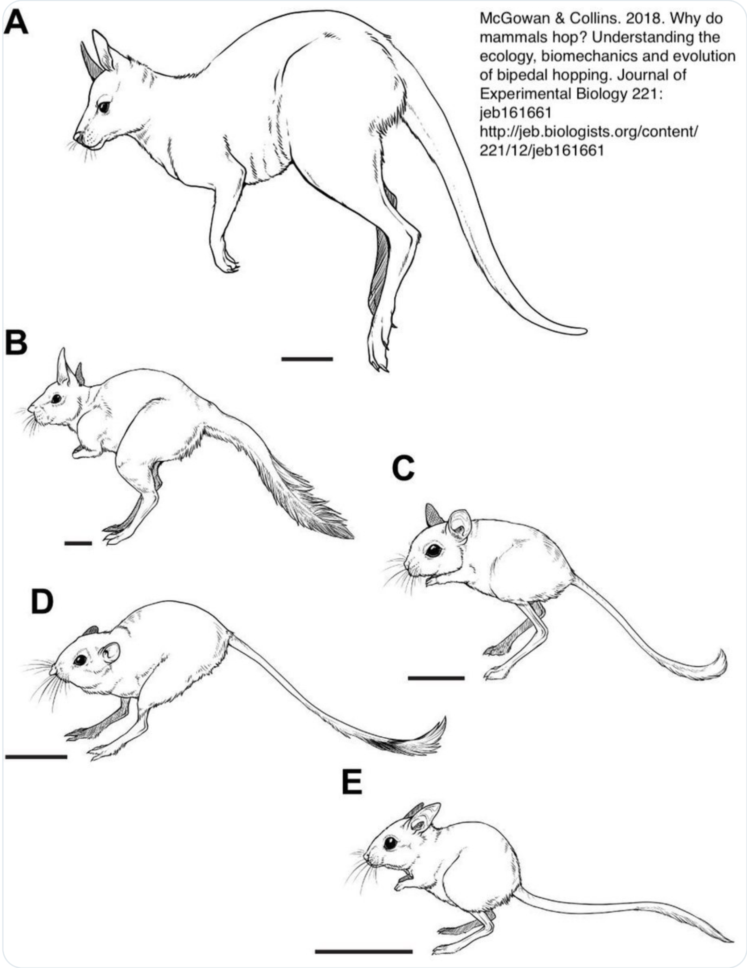

Most saltatorial mammals have specialized adaptations that allow them to be good & energetically efficient at jumping, such as elongated hindlimbs & long tails (kind of like kangaroos; see images attached to this & previous tweets in this thread) #2019MMM

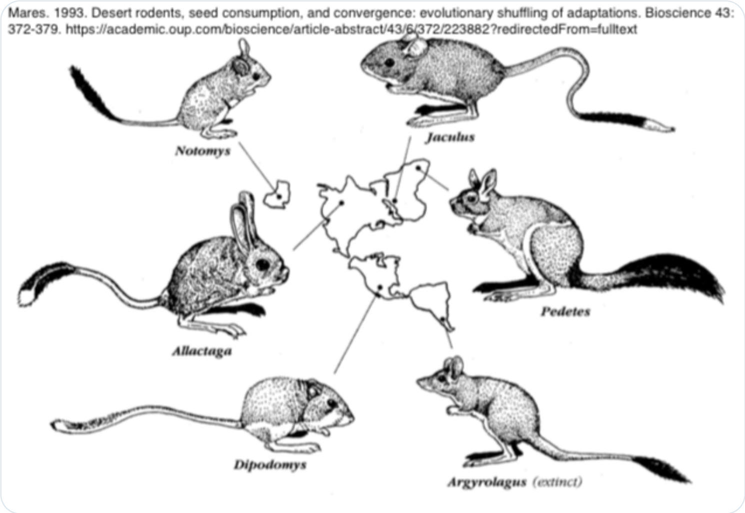

The hindlimbs in both the Springhare & Jackrabbit are elongated, as you can see

from these museum specimens from the Biodiversity Research and Teaching Collections at Texas A&M (specimens not scaled; just look at their hind legs) @wfsctamu #collectionsareessential #2019MMM

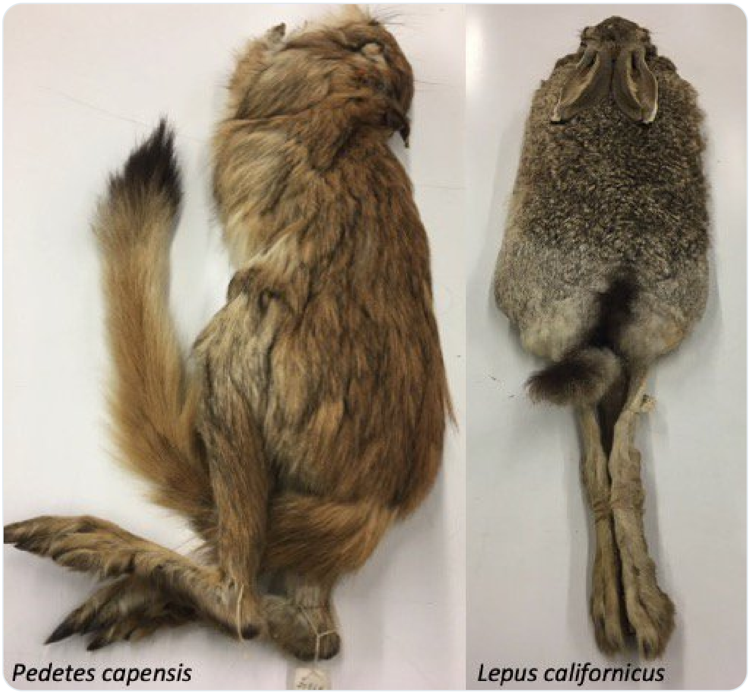

Do most of these saltatorial mammals look like kangaroos when they jump? Why yes, yes they do. Here's a Springhare example:

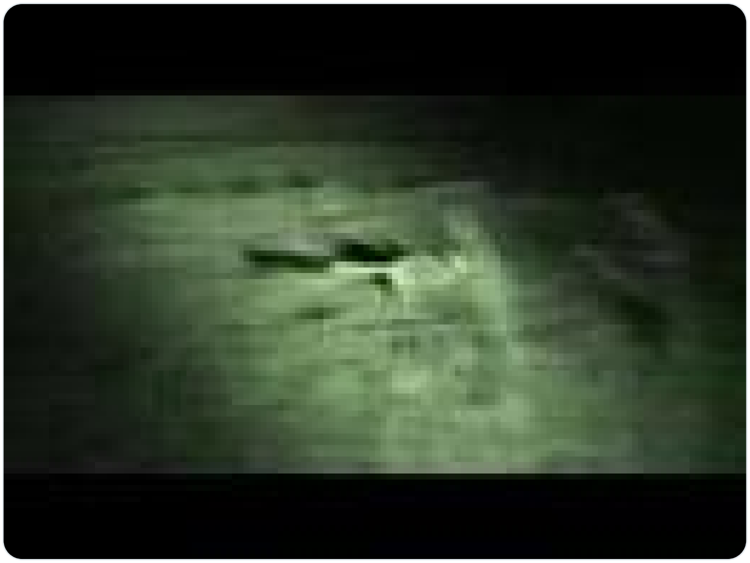

https://www.youtube.com/embed/hJ6IXRf44gA

#2019MMM

Many animals that are saltatorial often occur in wide open spaces, like deserts. Both Springhares & Jackrabbits have distributions that encompass desert habitats #2019MMM

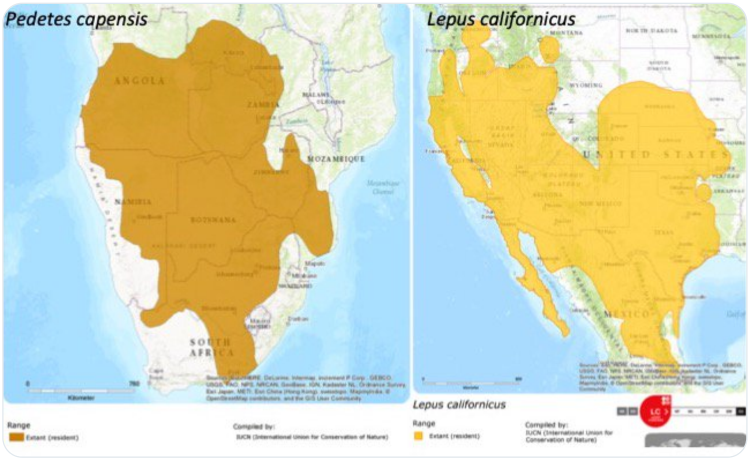

Desert animals often have additional adaptations to help them survive in these harsh environments. Look at the bottom of these Springhare & Jackrabbit feet! The fur helps with friction & provides insulation against the hot desert sand #2019MMM

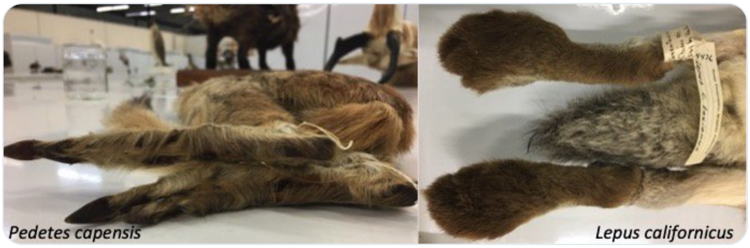

Our battle tonight takes place at Kruger National Park in South Africa https://en.wikipedia.org/wiki/Kruger_National_Park #2019MMM

It's an overcast & still night; perfect for our nocturnal combatants. Our Springhare is foraging near its burrow for roots & bulbs. Although generally solitary, Springhares often forage in groups because good food resources are usually clumped together (Butynski 1984) #2019MMM

Group foraging also comes in handy when its mating season, which it is pretty much year-round for Springhares. While foraging, our male Springhare spies a receptive female #2019MMM

Jackrabbits, depending on their location, also breed year-round. Our Jackrabbit is nearby, foraging for grasses & assessing the novel habitat, flora, & fauna, including the Springhares #2019MMM

Springhares are generally non-aggressive. But this stranger (that sort of looks like a Springhare) is a little too close to both the Springhare burrow & his potential mate (Anderson 1996; Laird 1993). Anger & aggression begin to build up in our Springhare #2019MMM

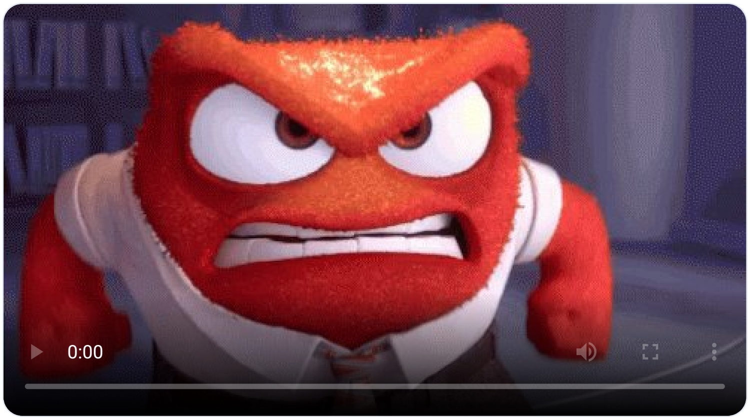

Sensing a bit of danger, Jackrabbit raises its ears attentively (Best 1993) #2019MMM

Springhare jumps at Jackrabbit

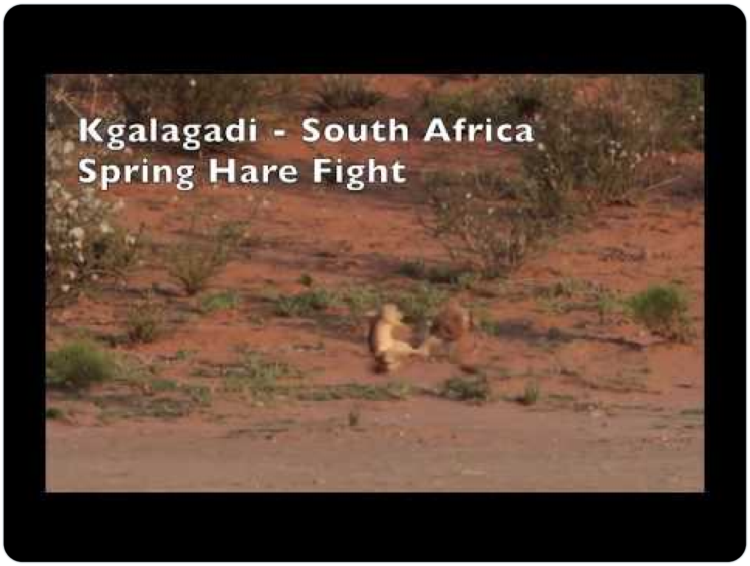

https://www.youtube.com/embed/GpN0c5MJ-fA

#2019MMM

Jackrabbit is startled, jumping 30-60 cm in the air (Best 1993). But Jackrabbit is not one to sit back a take a beating; he rears up on his hind legs

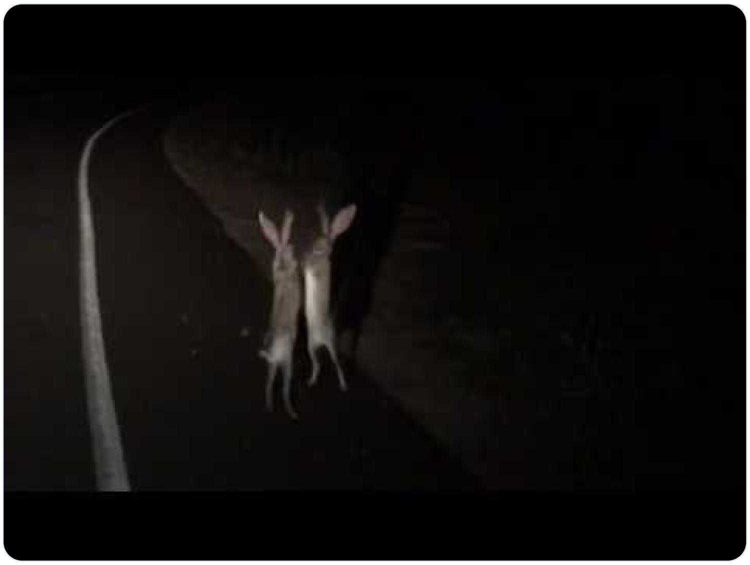

https://www.youtube.com/embed/H7CXQ4kXI40

#2019MMM

Jackrabbit forelimbs are bit longer than Springhare, & he is able to get in some good punches & bites, aiming for the ears of the Springhare (Best 1993) #2019MMM

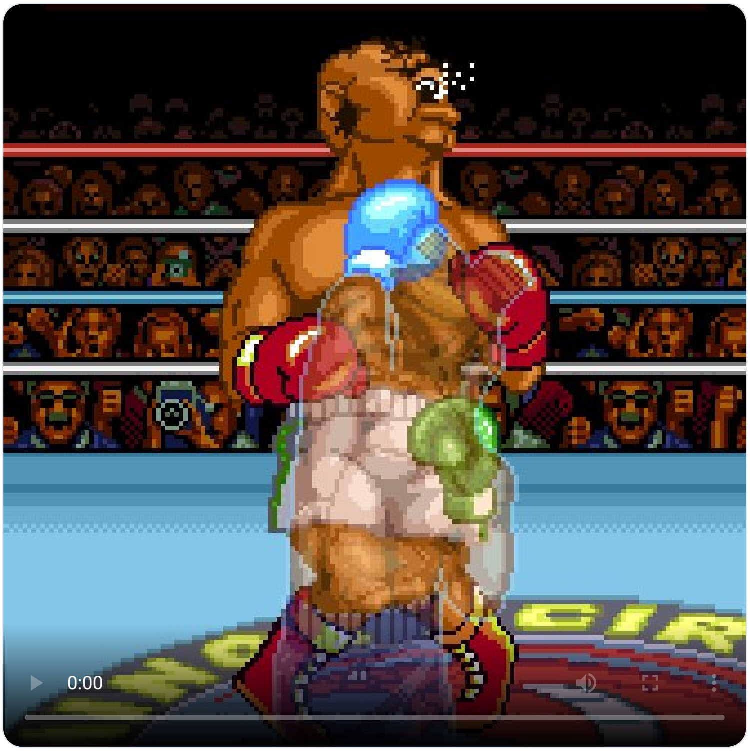

Spinghare leaps up, & lands big feet first on the Jackrabbit, all 4kg of its frame crushing the Jackrabbit #2019MMM

SPRINGHARE TROUNCES JACKRABBIT #2019MMM

• • •
