# Supplementary material for: March Mammal Madness and the power of narrative in science outreach
Source: eLife. 2021 Feb 22;10:e65066. doi: 10.7554/eLife.65066 (PMC7899649; doi:10.7554/eLife.65066)
Supplement: Supplementary file 3. [file elife-65066-supp3.pdf]

EXAMPLE SPORTS SUMMARIES 2018

Great Adaptations Round 2

1st seed Pygmy Hippo vs. 9th seed Maned Rat: DISPUTES OVER DINNER
In the first battle of the night we found the hippo and maned rat searching for dinner along the same stream in Sierra Leone. The maned rat didn't find the wetter climate to its liking, but soon found its attention turned to the hippo. The hippo had arrived to forage for fruits and ferns on its 5-6 hour feeding bout. On edge, the rat piloerected to show his poison bits, but the hippo presented a big challenge at a weight of 180-275 kgs (as opposed to the rat, which weighs in around 2-3 kg). The rat wisely decided that one challenge was enough for the day and forfeited the grounds to the hungry, hangry hippo to forage in another spot. Pygmy hippo advances. Narration by Patrice Connors.

5th seed Water Deer vs. 4th seed Tasmanian Devil: BEDEVILED
Though the diurnal water deer is at home in the coastal plains and marshy areas of China and Korean, this battle found us in the Tasmanian woodlands. The solitary deer happened upon the grisly sight of the Tasmanian devil munching on the carcass of a kangaroo. Naturally, the water deer let out a scream (or a "whicker") and snarls to show his saber canines. The Tasmanian devil answered with a scream of his own and the deer, which usually tries to avoid a fight, did just that and hopped off. Tasmanian Devil advances. Narration by Marc Kissel.

6th seed Jaguarundi vs. 3rd seed Crabeater Seal: SQUASH THE COMPETITION
The battle took place on the pack ice of the Southern Ocean, providing the Crabeater Seal with home court advantage. Jaguarundi got the drop on the seal by smelling his crustacean breath, but didn't have the advantage for long. Though the seal was unaccustomed to meeting a threat out of the water, the home court advantage worked in his favor. The jaguarundi lost the element of surprise due to its dark colored coat against the ice, but pounced anyway. Even with 9.7 mm teeth, however, the jaguarundi was unable to pierce the seal's 5 cm thick blubber and soon found that his hopes - and himself - squashed. Crabeater Seal advances. Narration by Josh Drew.

10th seed Aye-Aye vs. 2nd seed Cheetah: AYE DIDN'T SEE THAT ONE COMING
We returned to the Serengeti plains at sunset. The nocturnal aye-aye can travel up to 4 km a night by ground and tree. Though it was previously thought that Cheetahs would mostly stay put at night, Anne Hilborn discovered that they can move a lot at night as well. This evening, the cheetah decided to seek a better vantage point in a tree where he proceeded to startle himself and the aye-aye (in the middle of his dinner, no less). Both combatants fell out of the tree; the aye-aye snapped at the intruder and made the mistake of turning his back on the cheetah, who promptly bit off his head. Cheetah advances. Narration by Anne Hilborn.

Urban Jungle Round 2:

1st seed Harar Hyena vs. 9th seed Bristol Fox: WHO'S LAUGHING NOW
At 218 stoats (or 48kg) our female hyena was more than 3x times heavier than our male fox at 63 stoats (or 14kg). Our combatants faced off in the streets of Harar. The fox was confused about

how he ended up on these Ethiopian city streets but spied a meaty cow femur. Unfortunately for him, the hyena also spied that bone, and though the fox immediately took up a submissive stance the hyena decided not to let sleeping dogs (or foxes) lie. She grabbed him by the scruff to show him who was boss ... and broke his neck. Harar Hyena advances. Narration by Asia Murphy.

12th seed Sewer Rat vs 4th seed Coyote: PAINT THE TOWN… RED
These combatants faced off in Chicago, where the coyote's diet consists mainly of ... you guessed it: rats! Our sewer rat found himself cornered in a dark alley and leapt out of the way of the coyote's first attack. Coyotes miss 2/3 of the time, so this particular coyote was undeterred. The rat made a run for it with the coyote in hot pursuit, but took a wrong turn and found himself at a DEAD end. With a "shrieking vocalization" the desperate rat jump-attacked the coyote's head. The coyote counterattacked with a pounce of his own, however, and the rat was no match for his larger opponent. Coyote advances. Narration by Katie Hinde & Jessica Light

7th seed Porcupine against 2nd seed Cape Town Baboon: #COMEATMEBRO
This battle found us back in Cape Town, where our resident baboon had just finished a meal of hedgehog (RIP Sonic). The little critter left a bad taste in our baboon's mouth, so he set out looking for water. The water crisis led the baboon into the richer part of the city in search of a pool. He found one, but it had already been claimed by an overheated and unhappy porcupine. The baboon was unfazed since this porcupine was smaller than a Cape porcupine; unfamiliar with baboons, the porcupine sized him up at roughly the size of a young cougar. Determined, the porcupine put himself between the baboon and the water and exposed his barbed quills (and a warning odor). Porcupine called the baboon's threat-yawning bluff and slapped him in the face with a tail full of quills to win the day. Porcupine advances. Narration by Mauna Dasari.

6th seed Bobcat vs 3rd seed Berlin Boar: BOAR-ED
At roughly 12kg (60 stoats) the male bobcat was large, but the female boar had him beat at roughly 100kg (454 stoats). Our boar had just given birth to piglets, which made her behavior "increasingly protective and unpredictable" and also drove her to forage to sustain lactation. The bobcat approached stealthily and pounced the sleeping piglet pile, but their squeals of fear masked momma's return. At an impressive 25 miles an hour, the momma boar charged into the bobcat and biting down on one of his haunches. Infuriated, the bobcat slashed one of the sow's eyes with his switchblade claw. Momma boar flung the bobcat… into a graffitied wall. Berlin Boar advances. Narration by Katie Hinde, Mauna Dasari, Anne Hilborn, & Jessica Light
