# Supplementary material for: March Mammal Madness and the power of narrative in science outreach
Source: eLife. 2021 Feb 22;10:e65066. doi: 10.7554/eLife.65066 (PMC7899649; doi:10.7554/eLife.65066)
Supplement: Supplementary file 4. [file elife-65066-supp4.docx]

**March Mammal Madness in the Classroom:**

**Research the Contenders!**

**
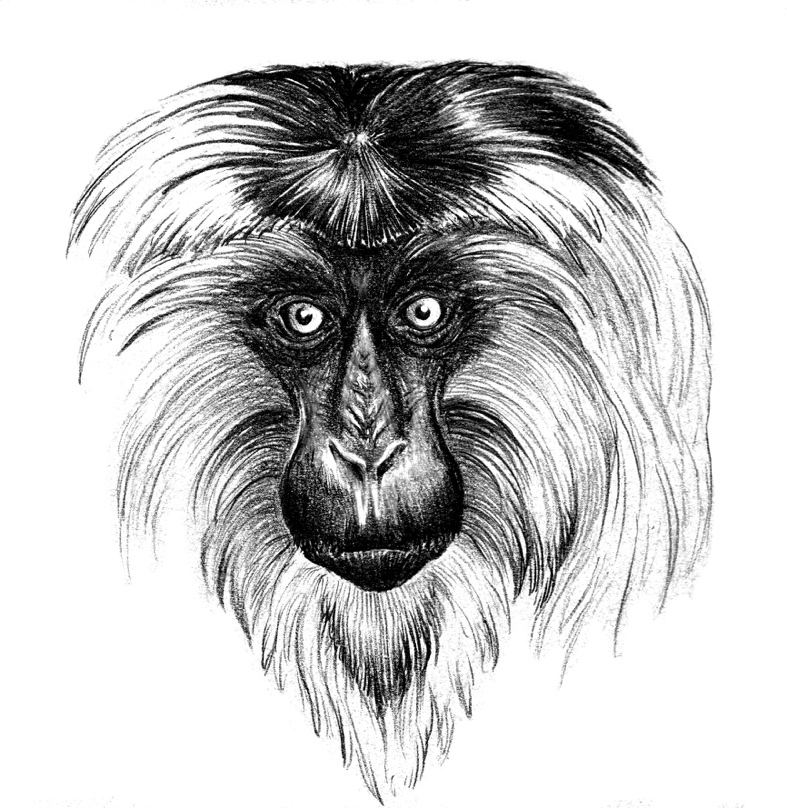
**

*March Mammal Madness is created and directed by Professor Katie Hinde, Arizona State University*

*Lesson plan by Dr. Stephanie Schuttler and Katie Hinde, artwork by Charon Henning*

**Objectives:**

- Students create species profiles of MMM “combatants” identifying adaptations that will help them or hurt them in battle
- Students fill out MMM personal brackets

**Timing and Implementation:**

- Variable. At least 10-15 minutes, but can be made into a full classroom activity or take place over several days for a few minutes each day
- Students must complete and share info before March 11^th^ (date of MMM “wild card” battle) or March 13^th^ (launch date of the first round)

**Materials:**

- MMM bracket
- Google drive and Internet

**Introduction:**

Mammal March Madness (MMM) is an alternate March Madness tournament focusing on simulated encounters between non-human mammals (and sometimes non-mammal animals and now in 2019… plants!), instead of college basketball. The bracket is run by a team of evolutionary biologists, using science and probability to determine the outcomes of the “battles.” The goal of Mammal March Madness is to provide a fun and exciting way to spread scientific knowledge about the competing animals, as well as awareness of ecology and animal conservation. These lesson plans are designed to extend learning of MMM in the classroom aligning to content required by state standards- particularly traits/adaptations, evolutionary relationships, anatomy and physiology, ecosystems.

For more info, visit: <https://libguides.asu.edu/MarchMammalMadness> <https://en.wikipedia.org/wiki/Mammal_March_Madness>, and <https://theconversation.com/march-mammal-madness-tournament-shows-the-power-of-performance-science-73425>

**Procedure:**

In 2019 there are 82 species participating in MMM, many of which you and your students are unlikely to have ever even heard of! Typically the tournament is 65 species (16 species per division, plus an additional wild card competitor). In 2019 we have included the Tag Team division in which mutualists compete as a team, increasing the number of featured species. For students to make better decisions on which species should win each battle, they will create species profiles as a class using the Arizona State University’s resource page: <https://libguides.asu.edu/MarchMammalMadness> (websites under “Pick your Winners”).

1. Create a shared Google Drive folder for students to contribute to. For each species/team participating in MMM, create a blank document within the folder.
2. Briefly discuss what is a mammal. Ask students what are the characteristics unique to mammals and how they differ from other animals.
3. Assign each student a species to research for “vital stats” (see #4 below). For example, in a class of 32 students, each student will be assigned (or choose) two different species. Each student must choose a different species or not all of the species will be properly researched. Because we have such a large number of species in 2019, teachers may consider asking students to select from a subset of competitors for their research.
4. Briefly review the meaning of adaptations/traits. For each species a student researches, they must put the following information in the google drive:
   1. 1 photo
   2. 1 structural adaptation the animal has that is a strength (structural adaptations = weaponry, armor, camouflage, nutrition, respiration, transport and excretion mechanisms, movement)
   3. The biome the animal lives in
   4. If the species is an herbivore, omnivore, or carnivore
   5. The trophic level of the species (primary, secondary, or tertiary consumer).
5. The students will then advocate (or not, depending) for their animal to be a fierce competitor in MMM. They can do this just for their class or for all of #2019MMM participants to learn about the various species through Twitter. Lots of people participating will not know these species and these videos/tweets will be really helpful to people. They will share the information by:
   1. Brief presentations to class
   2. Sharing info in a tweet using the hashtag #2019MMM and #2019MMMK12
6. Students listen to information from their classmates and/or use tweets (from around the nation!) to make their choices and fill out their brackets. Note – students do not have to choose the species they researched to win. Decide what the student with the most points wins!
7. Additional/Alternative Task: Students can do a promotional poster for their predicted finals highlighting what traits will carry them to the Championship “battle”! Examples: https://twitter.com/aminakatanaa/status/973005996464529408 https://twitter.com/search?f=tweets&q=%232018MMMK12&src=typd

**March Mammal Madness in the Classroom:**

**Let the Games Begin!**

**
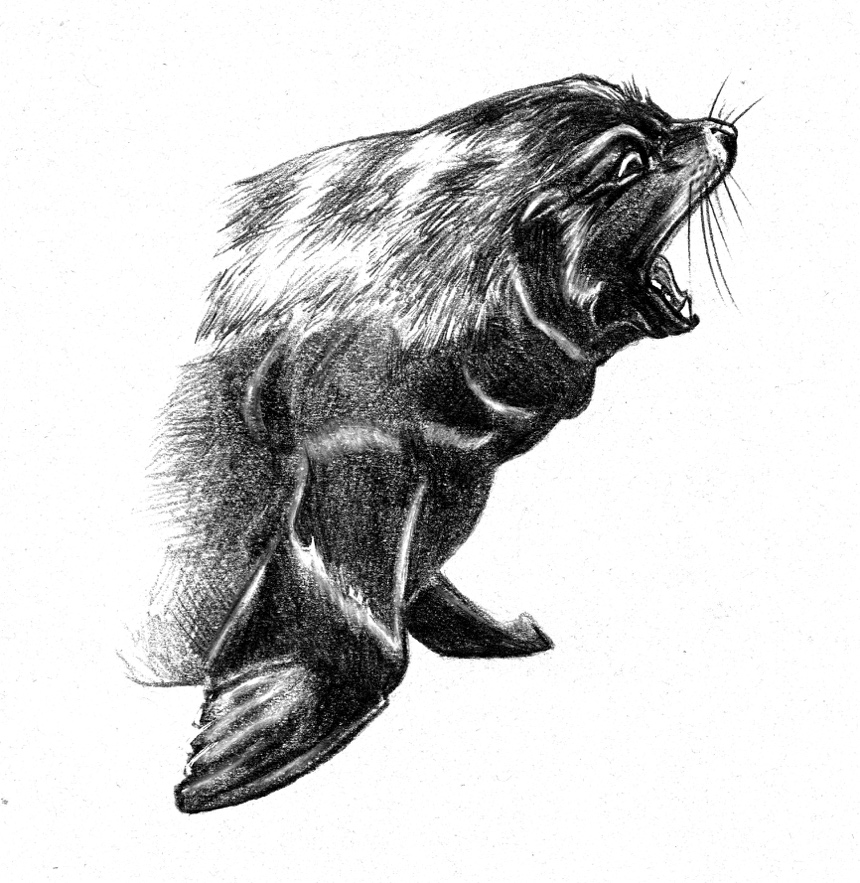
**

*March Mammal Madness is created and directed by Professor Katie Hinde, Arizona State University*

*Lesson plan adapted from Dr. Stephanie Schuttler and Katie Hinde, artwork by Charon Henning*

**Objectives:**

- Students learn about the species through tweeted-out competitions conducted by scientists from universities around the nation
- Students communicate directly with scientists through Twitter using #2019MMM during competitions
- Students update winning species with each round, adding information on behavioral adaptations, environmental impacts, human impacts, and knowledge on species evolutionary history.

**Timing and Implementation:**

- Variable, but least ~15 minutes at night, 10-15 minutes in the morning

**Materials:**

- Worksheets
  - #2019MMM: Round 1 – Behavioral Adaptations (4 copies)
  - #2019MMM: Round 2 – Traits & Scientist Profiles (2 copies)
  - #2019MMM: Round 3 – Sweet 16 Human Impacts (1 copy)
  - #2019MMM: Round 4 – Elite Trait – Environmental Impacts (1 copy)
  - #2019MMM: Round 5 – Final Roar – Evolutionary History (1 copy)
  - #2019MMM: Round 6 – Championship
- Google drive and Twitter
- Depending on time zone, some students may not be able to stay up to watch the “battles” live tweeted- the key battle tweets will be available the next morning at wakelet: https://wakelet.com/@ChrisAnderson2426

**Procedure:**

1. There are N=32 1st round battles (eight per Division) that are play-by-play announced via twitter (each battle has 15-25 tweets presenting species & battle information prepared by the biologist narrators), N=16 2^nd^ round battles (4 per Division), N=8 3^rd^ round battles (the Sweet 16), N=4 4^th^ round battles (the Elite Trait), N=2 5^th^ round battles (the Final Roar) and the Final Championship. Rounds 1-3 the “battle” occurs in the better seeded species home ecology- they have home court advantage. Rounds 4-6, the battle takes place in a randomized location announced at the start of the battle. In 2019 the randomized locations are Mangrove, Sandhills, Thorn Forest, and Lake. Students are encouraged to follow tweets from the account <https://twitter.com/2019MMMletsgo> (@2019MMMletsgo) to learn more about the contestants and discover the winners of the battle. Please note that there are official contributors and thousands of fans using the tournament hashtag. For educators who want the streamlined information about the battle and/or assuredly safe content for the classroom context, then they should follow the @2019MMMletsgo because this account only retweets the official tournament tweets.
2. Each night of the battles, the students fill out the attached worksheets in relation to their bracket picks. Each round will have a different focus. The following day, the students will report back to the teachers and have a discussion based on their worksheets.

***Round 1 – Physical Traits***

Students summarize physical traits of the animals announced by the battle narrators, especially those that played a role in the outcome.

***Round 2 – Behavioral Adaptations & Scientist Profiles***

Students describe key behavioral adaptations of the species announced by the battle narrators, especially behaviors that caused the battle outcome, and look up information about one scientist/researcher highlighted in one of the battles

***Round 3 Sweet 16 –Human Impacts***

Students look up IUCN conservation info about the winner of each battle, summarizing conservation status, threats, and describe current or possible conservation efforts.

***Round 4 Elite Trait – Environmental impacts***

In round 4, the habitats that animals will battle in will be chosen at random from 4 different ecologies: mangrove, sandhills, thorn forest, lake. Students summarize how the environment did or could have played a role in the battle outcome by favoring or disfavoring one or both combatants in each battle.

***Round 5 – Final Roar - Evolutionary history***

Students summarize the evolutionary history of the winner and using report when competitors in each match-up last shared a common ancestor using TimeTree.org.

***Round 6 – Final Championship***

Students compare and contrast the #2019MMM Champion with the species they had thought would win the tournament.

**PERMUTATIONS:**

Option A: Specific worksheets for each division round 1 that emphasize anatomy & physiology (JUMP JUMP), aquatic ecosystems (WATERFALLS), classification system (CAT-e-GORY), and mutualisms (TAG TEAM)

Option B: Genetics Info Worksheet
