# Supplementary material for: March Mammal Madness and the power of narrative in science outreach
Source: eLife. 2021 Feb 22;10:e65066. doi: 10.7554/eLife.65066 (PMC7899649; doi:10.7554/eLife.65066)
Supplement: Supplementary file 5. [file elife-65066-supp5.docx]

**#2019MMM: Round 1 – Traits**

Division:_____________________________________________

| Battle  (Species vs. Species) | Which species did you predict would win? | Which species won? | New fun fact you learned about species that lost: | New fun fact you learned about species that won: | What is a physical trait that may help the winning animal win again? |
| --- | --- | --- | --- | --- | --- |
|  |  |  |  |  |  |
|  |  |  |  |  |  |
|  |  |  |  |  |  |
|  |  |  |  |  |  |
|  |  |  |  |  |  |
|  |  |  |  |  |  |
|  |  |  |  |  |  |
|  |  |  |  |  |  |

*Physical trait examples – body size, teeth (canines! carnassials!), antlers, tusks, reach, temperament, etc.*

**Name:________________________**

**#2019MMM: Round 2 – Behavioral Adaptations & Scientist Profiles**

Divisions:_____________________________________________

| Battle  Species vs. Species  (circle winner) | Did the winner have a behavioral trait that was important for their win? (If yes, describe the trait) | If any scientists and their research were featured in the battle, list their Name, University, & Research Topic |
| --- | --- | --- |
|  |  |  |
|  |  |  |
|  |  |  |
|  |  |  |
|  |  |  |
|  |  |  |
|  |  |  |
|  |  |  |

*Behavioral adaptation examples – hunting behaviors (like predatory stalking, ambush), social behaviors, migration , parental care, food processing, learning, social structure of the species (e.g. cooperative group-living)*

**Name:________________________**

**#2019MMM: Round 3 – Sweet 16 – Human Impacts**

| Battle  Species vs. Species (circle winner) | What is the IUCN red list conservation status of the winning species? | What are the biggest threats to the winner? | For threatened and endangered species, what conservation programs are or could be implemented for protection? |
| --- | --- | --- | --- |
|  |  |  |  |
|  |  |  |  |
|  |  |  |  |
|  |  |  |  |
|  |  |  |  |
|  |  |  |  |
|  |  |  |  |
|  |  |  |  |

**Name:________________________**

**#2019MMM Round 4 – Elite Trait – Environmental impacts**

| Battle  Species vs. Species (circle winner) | What is a specific abiotic factor that helped the winner? (e.g. sea ice for polar bears) | Which randomly chosen ecosystem would have been the worst for the winner? Explain why. |
| --- | --- | --- |
|  |  |  |
|  |  |  |
|  |  |  |
|  |  |  |

**Name:________________________**

**#2019MMM: Round 5 – Final Roar – Evolutionary history**

| Battle  Species vs. Species  (circle winner) | When did the combatants last share a common ancestor? (use timetree.org) | Is the winning species a generalist or a specialist? Explain your answer. | What is a closely related species to the winner? |
| --- | --- | --- | --- |
|  |  |  |  |
|  |  |  |  |

**Name:________________________**

**#2018MMM: Round 6 – Championship!!**

| Who did you predict to be champion? | Who was the official Champion? | Based on everything you know about the Champion, do you think it should have won March Mammal Madness? Why or why not? | Do you still think the species you picked should have been the winner? Why or why not? |
| --- | --- | --- | --- |
|  |  |  |  |

Which species do you want to see in #2020MMM? (Tweet it out!): _______________________________________

**Name:________________________**

**ALTERNATE ROUND ONE WORKSHEETS SPECIFIC TO DIVISIONS**

(may be particularly useful for High School AP classes)

These worksheets emphasize anatomy & physiology, ecosystems,

classification system, and mutualisms

**#2019MMM: Round 1 – WATERFALLS**

| Battle  Species vs. Species  (circle winner) | Which species did you predict would win? | For the species that “lost”: what type of water ecosystem do they live in & what is an adaptation they have for that water ecosystem? | For the species that “won”: what type of water ecosystem do they live in & what is an adaptation they have for that water ecosystem? |
| --- | --- | --- | --- |
|  |  |  |  |
|  |  |  |  |
|  |  |  |  |
|  |  |  |  |
|  |  |  |  |
|  |  |  |  |
|  |  |  |  |
|  |  |  |  |

**Name:________________________**

**#2019MMM: Round 1 – Jump Jump**

| Battle  Species vs. Species  (circle winner) | Which species did you predict would win? | For the species that “lost”: describe their jumping behavior & physical traits | For the species that “won”: describe their jumping behavior & physical traits |
| --- | --- | --- | --- |
|  |  |  |  |
|  |  |  |  |
|  |  |  |  |
|  |  |  |  |
|  |  |  |  |
|  |  |  |  |
|  |  |  |  |
|  |  |  |  |

*Jumping Behavior examples– vertical cling & leap, stotting, distance/height; physical trait examples – aspects of anatomy such as limb length and/or musculature; health and energy status*

**Name:________________________**

**#2019MMM: Round 1 – CAT-e-GORY**

| **Battle**  Species vs. Species  (circle winner) | For the species that “lost”: what is the actual animal* group it belongs to and what trait does it have that the species was named for a “cat” | For the species that “won”: what is the actual animal* group it belongs to and what trait does it have that the species was named for a “cat” |
| --- | --- | --- |
|  |  |  |
|  |  |  |
|  |  |  |
|  |  |  |
|  |  |  |
|  |  |  |
|  |  |  |
|  |  |  |

*All species in the CAT-e-GORY Division have something about “cat” in their common or Latin name, but none of them are in the felidae. *one plant combatant in this division- DandeLION*

**Name:________________________**

**#2019MMM: Round 1 – TAG TEAM**

| **Battle**  Species vs. Species  (circle winner) | For the team that “lost”, describe their mutualism. | For the team that “won”, describe their mutualism |
| --- | --- | --- |
|  |  |  |
|  |  |  |
|  |  |  |
|  |  |  |
|  |  |  |
|  |  |  |
|  |  |  |
|  |  |  |

**Name:________________________**

**#2019MMM: Genetics Facts**

Follow Geneticists Professors Anne Stone @StoneLab_ASU & Melissa Wilson @sexchrlab

Each battle they tweet genetics & phylogeny info about the competitors

(& an RIP tweet after a combatant loses)

**Name: _______________________________ Division: _________________ Round: _________________**

| **Battle**  Species vs. Species  (circle winner) | **Describe what you learned from the genetics fun facts & RIP tweet for the competitors** |
| --- | --- |
|  |  |
|  |  |
|  |  |
|  |  |
|  |  |
|  |  |
|  |  |
|  |  |
