# Supplementary material for: March Mammal Madness and the power of narrative in science outreach
Source: eLife. 2021 Feb 22;10:e65066. doi: 10.7554/eLife.65066 (PMC7899649; doi:10.7554/eLife.65066)
Supplement: Supplementary file 6. [file elife-65066-supp6.pdf]

#2018MMM Early Bracket Request for Educators

WELCOME EDUCATOR!!! Thank you & your students for participating in, as one twitter user phrased it, this "delightfully bonkers" science education outreach. The organizers and contributors of March Mammal Madness really appreciate you sharing this game with your students and helping increase knowledge, joy, awe, and imagination around science and the natural world.

To facilitate your lesson planning, below you can submit your email address to request the bracket (and other tournament information) in advance of the public release. The bracket and materials will be emailed to you on Feb 20 (the bracket drops for the general public on March 1) and the tournament officially begins with the wild card battle on March 12.

Further below, I am also inviting your participation in a brief 10-minute survey "Educator Use of Online Science Outreach: March Mammal Madness Simulated Animal Tournament" so I can better understand how educators use this tournament in the classroom. I am a professor in the Center for Evolution and Medicine at Arizona State University. I am conducting a research study to better understand how teachers use March Mammal Madness in their classrooms.

This survey is totally optional and your participation is voluntary. You have the right not to answer any question, and to stop participation at any time. AND importantly whether or not you participate has no effect on advance access to the materials. You must be 18 or older to participate in the study. There are no foreseeable risks or discomforts to your participation. Your responses will be anonymous. The results of this study may be used in reports, presentations, or publications, but your name will not be used.

If you have any questions concerning the research study, please contact me at Katie.Hinde@asu.edu. If you have any questions about your rights as a subject/participant in this research, or if you feel you have been placed at risk, you can contact the Chair of the Human Subjects Institutional Review Board, through the ASU Office of Research Integrity and Assurance, at (480) 965-6788. Filling out the survey will be considered consent; individuals who do not want to participant can exit the survey after submitting their email address to request early access to materials.

#2018MMM

1. Email Address

2. Class(es) You Teach

3. Grade Level(s)

____

4. Local Community

Mark only one oval.

- ◯ Rural
- ◯ Suburban
- ◯ Urban
- ◯ Other: _______________

5. Geographical Region

Mark only one oval.

- New England & Northeast (MA, RI, VT, NH, ME, CT)
- Mid-Atlantic (PA, NY, NJ)
- Great Lakes (WI, IL, IN, OH, MI)
- Midwest Plains (ND, SD, NE, KS, MN, IA, MO)
- Mountain (CO, UT, WY, NV, MT, ID)
- Pacific (OR, WA, CA, HI, AK)
- South Atlantic (FL, GA, SC, NC, VA, DC, WV, DE, MD)
- Southwest (AZ & NM)
- West South Central (LA, OK, TX, AR)
- East South Central (MS, KY, TN, AL)
- Canada & Mexico
- Central & South America
- Sub-Saharan Africa
- Middle East & North Africa
- Central Asia
- Australia & Pacific Islands
- South Asia & Southeast Asia
- Europe

6. How many years have you been using Mammal March Madness in teaching?

Mark only one oval.

- This is my first year
- 2-3 years
- 4-6 years

7. How do you use MMM in your classroom?

____

____

____

____

____

8. In 2018, how many students will you distribute MMM materials to?

____

9. Prior to 2018, estimate the number of the students whose participation in MMM you facilitated:

____

10. Please add any comments you wish to share about MMM

This content is neither created nor endorsed by Google.

Google Forms
